# Protocol for a systematic review and meta-analysis of observational studies examining the impact of COVID-19 safety measures on physical activity patterns in adults

**DOI:** 10.1186/s13643-021-01818-y

**Published:** 2021-10-29

**Authors:** Fabian Schwendinger, Denis Infanger, Elena Pocecco, Joséphine Gander, Timo Hinrichs, Arno Schmidt-Trucksäss

**Affiliations:** 1 Division of Sports and Exercise Medicine, Department of Sport, Exercise and Health, University of Basel, Grosse Allee 6, 4052 Basel, Switzerland; 2 Department of Sports Science, Medical Section, University of Innsbruck, Fürstenweg 185, 6020 Innsbruck, Austria

**Keywords:** *S*ARS-nCOV-2, Coronavirus, Social distance, Sedentary behavior, Locomotion, Physical inactivity

## Abstract

**Background:**

The primary objective of this study is twofold: (1) to examine the effect of COVID-19 safety measures, enacted to prevent transmission of SARS-nCOV-2, on total physical activity in the adult general population (≥ 18 years) and (2) to analyze the impact of the factor “severity of safety measures” on potential changes in physical activity. The secondary objective is to investigate the effects of safety measures on the respective PA intensities, i.e., sedentary behavior, light, moderate, and vigorous physical activity.

**Methods:**

A systematic literature search will be performed in the following online databases: Medline (on Ovid), Web of Science, Scopus, L^.^OVE Coronavirus disease by Epistemonikos, and ProQuest Dissertations & Theses A&I. All obtained citations will undergo title and abstract as well as full-text screening by two independent reviewers. Observational studies investigating the effects of safety measures on physical activity patterns in the adult general population will be included. The standardized mean difference in total physical activity per time unit between pre- and during COVID-19 or between normative data and during COVID-19 will be the primary outcome. The standardized mean difference in sedentary time, light, moderate, and vigorous physical activity will be assessed as secondary outcomes. Eligible studies will be divided between the reviewers for data extraction using a pilot-tested data form. Risk of bias assessment will be performed using a standard assessment tool. If suitable, a random-effects meta-analysis and meta-regression with a unit of safety measure severity as the independent variable will be performed.

**Discussion:**

This study will synthesize available data reporting the effect of COVID-19 safety measures on physical activity patterns in adults. Furthermore, we will incorporate a unit for the severity of safety measures for better generalizability of the results. These findings will be of great value for public health policymaking and estimating future health consequences.

**Systematic review registration:**

PROSPERO CRD42021231039.

**Supplementary Information:**

The online version contains supplementary material available at 10.1186/s13643-021-01818-y.

## Background

### The scenario

The coronavirus disease 2019 (COVID-19), initiated by the severe acute respiratory syndrome coronavirus 2 (SARS-nCOV-2), has developed into a worldwide pandemic causing substantial physical as well as psychological health issues [1, 2]. To contain the spread of SARS-nCOV-2, authorities have implemented safety measures such as social distancing, the shutdown of public facilities, lockdowns, or home quarantine, largely restricting daily life. The extent of these measures has in most countries been determined by the number of new COVID-19 infections in a certain period and varies accordingly between countries and even regions or cities. As an example, Switzerland, Germany, and Austria enacted strict lockdowns at the beginning of the pandemic [3]. With the number of new infections decreasing in summer, the safety measures have been mitigated, enabling the reopening of shops and public facilities (i.e., gyms, sports clubs, parks) [3]. This shows how public life was and still is impacted by the constantly changing COVID-19 situation.

### The root of a great burden for the healthcare systems in the future

Already during the first COVID-19 wave (approximately from December 2019 until the middle of 2020), our research group and many others began to draw attention to the arising problem of reduced physical activity (PA) [4–6]. PA is an important modifiable risk factor for numerous chronic diseases and all-cause mortality [7]. Greater time spent physically active is independent of PA intensity associated with a substantially reduced risk of premature mortality [8]. On the other hand, pronounced physical inactivity is strongly connected to undesirable health outcomes such as chronic non-communicable diseases (including cardiovascular diseases) [7] and therefore an immense economic burden for healthcare systems [9]. According to conservative estimations for the year 2013, physical inactivity was responsible for healthcare costs of 53.8 billion $ worldwide [9]. Besides, it was recently shown that regular PA may act as a protective factor for COVID-19 and especially a severe disease course [5, 10, 11]. It is, therefore, highly relevant to examine potential changes in PA due to COVID-19 safety measures.

### The rationale for this study

Since the onset of the pandemic, numerous studies have been conducted around the globe highlighting changes in PA patterns due to the safety measures in place [12–15]. Both, studies using self-reported data [12, 15] and trials using objective methods (i.e., wearables or smartphones with accelerometers, pedometers, or global positioning systems) [13, 14] have researched this area. Available studies largely vary in their methodology when it comes to the assessment tool used, study population, country, or period in which the data collection was performed [12–15]. Such differences make it difficult to draw conclusions. To date, there is one systematic review available that aimed to examine changes in PA and sedentary time from before to during the COVID-19 lockdown (*n* = 45 studies in healthy adults) [16]. Yet, we feel that since the end of their search in June 2020, further important studies have been published that need to be taken into account. Finally, the discrepancies in safety measures between countries and the fluctuations in their extent since the outbreak of the pandemic make it difficult to transfer study results to real-life.

The planned study will, therefore, synthesize the available evidence from different countries and periods in time, making data more accessible and utilizable for public health policymaking and facilitating the development of adequate countermeasures. We will investigate whether the safety measures led to changes in PA patterns in the adult general population (≥ 18 years of age). Furthermore, this will likely be the first study taking the extent of safety measures into account.

## Methods/design

Before the onset of this project, we screened PubMed, L^.^OVE Coronavirus disease by Epistemonikos, and the International Prospective Register of Systematic Reviews database (PROSPERO), ensuring no systematic reviews or meta-analyses have been or are being conducted to this research question. To support transparent science, this protocol was registered and published in PROSPERO on 4 February 2021 (ID: CRD42021231039). All procedures of this project will follow the guidelines by Tawfik et al. [17]. The items in this systematic review protocol were reported according to the Preferred Reporting Items for Systematic Review and Meta-Analysis Protocols (PRISMA-P) guidelines (Additional file 1) [18, 19].

### Eligibility criteria according to PICOS

All applied eligibility criteria are listed below according to the PICOS scheme.

#### Population (P)

All trials on adult humans (≥ 18 years) of either sex in free-living condition will be eligible for inclusion with the following exceptions. Studies including only specific populations (i.e., athletes, patients, males, fitness app users) or adults that previously or acutely contracted SARS-nCOV-2 will be excluded as these would not accurately reflect the general population that was not directly affected by COVID-19. The presence of chronic non-communicable diseases in some but not all study participants will not be an exclusion criterion.

#### Intervention (I)

Eligible studies must investigate the effects of COVID-19 safety measures. This term was defined as any social or public measure implemented to prevent transmission of COVID-19, including social distancing, self-isolation, closure of public facilities, cancelation or restriction of public events and gatherings, travel and mobility restriction, and working from home [20].

#### Control (C)

Only studies with a control condition will be included. The control condition was defined as a measure of PA in the absence of any COVID-19 safety measure, e.g., data from study cohorts prior to the COVID-19 outbreak, normative data, or retrospectively gathered data.

#### Outcome (O)

PA must be assessed as a study outcome. PA was defined as any bodily movement by the skeletal musculature resulting in energy expenditure [21]. The assessment can be done via (online) questionnaires, diaries, interviews, or using wearable technology (i.e., pedometers, accelerometers, global positioning system, or mobile applications) but is not limited to these. However, the outcome must be quantified for instance in minutes, steps, distance covered, kilocalories burned, or acceleration per time unit. Dichotomous and categorical data (e.g., more/less PA) will be excluded from this study to achieve a higher accuracy of the data. Indeed, the most recent WHO guidelines on physical activity by Bull et al. [22] explicitly stated that every minute of PA counts. Moreover, as some countries might have only mild safety measures in place, changes in PA may not be very pronounced and would not be accurately measured using categorical data.

#### Study design (S)

Observational studies (longitudinal and cross-sectional) in English, German, French, and Italian language will be included. Studies in other languages will be eligible if a translation is available. Systematic reviews will be included as a source of potentially relevant primary studies. Experimental trials applying physical activity or exercise interventions will be excluded. Further exclusion criteria are unrelated, duplicated, or abstract-only papers, case reports and case series, editorials, and papers with no full-text available. Finally, only studies published after October 2019 will be eligible for inclusion.

### Information sources and search strategy

The search strategy was developed using the PICOS scheme described above and included database-specific controlled vocabulary used for the indexing of articles (such as medical subject headings in Medline), free-text terms (title and abstract), and subheadings. To search for studies on COVID-19 and safety measures, two search strings developed by the Canadian Agency for Drugs and Technologies in Health were adapted [20]. The search will be initiated in Medline (on Ovid) and later translated for Web of Science (core collection), Scopus, and L^.^OVE Coronavirus disease by Epistemonikos. We additionally search for gray literature in ProQuest Dissertations & Theses A&I (on proquest.com) to reduce publication bias (start of search: 18 April 2021). The full search strategy (available in Additional file 2) was peer-reviewed using the PRESS checklist by an information specialist of the Basel Medical University Library in Basel, Switzerland [23]. We are aware of the recommendation not to include outcomes in the search strategy as they are often poorly described in the title or abstract of articles [24]. Yet, for the planned study, it seems unavoidable. Based on preliminary searches and reviewing relevant studies, we are convinced that the terms used in the search strategy are adequately described in the literature. In addition to the systematic literature search, citation tracking (backwards and forwards) will be used to identify relevant literature. The searches will be re-run prior to final analyses to avoid overlooking any relevant citations.

### Data management

All citations obtained from the systematic searches of the selected databases will be exported to EndNote X9.3.3 or a later version (Clarivate, Philadelphia, USA), where deduplication will be performed. At this stage, the author’s names, publication year, journal, URL, and abstract will be extracted to a pilot-tested Excel data form that will be compiled based on Tawfik et al. [14].

### Screening procedures

Before the onset of the screening procedures, reviewers will be trained and evaluated based on Cohen’s kappa of agreement [24]. The reviewers will independently review a set of 50 randomly selected articles [25]. In the case of Cohen’s kappa < 0.8, training will be repeated [25].

All screening procedures will be performed independently by two reviewers (FS and EP) following the aforementioned inclusion and exclusion criteria, blinded for the other reviewer’s decisions. The process will be facilitated using the Rayyan web application for systematic reviews [26] and all decisions will be documented by denoting “yes,” “no,” or “maybe.” Citations that received the denotation “maybe” will be solved by consensus between the reviewers. In the case of no settlement, a third reviewer (JG) will be consulted. During the screening procedures, the deduplication process will continue manually. The process will start with title and abstract screening. Subsequently, the full-text will be obtained and all citations that have not yet been excluded will undergo full-text screening. During this step, all excluded citations will be given an exclusion reason [17]. Given no full-text is available, study authors will be contacted, the request full-text function on ResearchGate will be used, or it will be purchased if available. All decisions and exclusion reasons will be presented in a PRISMA flow diagram. Following the screening procedures, the inter-rater agreement will be documented using Cohen’s kappa of agreement [19].

### Data extraction

In this step, all included studies will be divided between the three reviewers (FS, EP, and JG) and the following data will be extracted independently into the Excel data form: study design including the country and period during which the study was conducted, sample size, type of comparison used (e.g., control group or normative data), inclusion and exclusion criteria, subject characteristics, data on the outcomes defined hereinafter, details on the method used to measure PA, and unit of measurement. A data checking step will be included following data extraction to prevent human error. Each reviewer will be assigned one-third of the included citations and compare them to the data collection form.

The primary study outcome will be the standardized mean difference in total PA (TPA) per time unit between pre- and during COVID-19 or between normative data and during COVID-19. The standardized mean difference in sedentary time and the intensity categories of PA (light [LPA], moderate [MPA], and vigorous [VPA] or moderate-to-vigorous [MVPA]) will be secondary outcomes. This will yield important additional information, as especially higher PA intensities are relevant for physical conditioning [22]. Efforts will be taken to transform relevant data into similar units (e.g., min per week to min per day). If no information about TPA is available, LPA, MPA, and VPA will be summed up to yield TPA. For studies investigating the effects of safety measures at several points in time, all relevant data will be extracted.

Supposing that data need to be extracted from graphs, Web plot digitizer [27] will be used to facilitate this process. To calculate missing variables during data extraction, previously reported equations will be used [28, 29]. Results only reported as medians will be transformed to means accordingly [29]. Missing standard deviations will either be calculated based on other available study information [28, 29] or standard deviations of similar studies will be imputed [30]. When missing data cannot be calculated, we will attempt to contact the authors of the publication to obtain the relevant missing data. The procured unpublished information will briefly be described in the manuscript. If studies only reported adjusted outcome values, they will be included according to the recommendations of the Cochrane Handbook for Systematic Reviews of Interventions [24]. To reduce the risk of bias through the inclusion of studies published more than once, we will compare author names, sample sizes, outcomes, and locations where the trials were conducted [19].

### Risk of bias assessment and data checking

Risk of bias assessment will be done by two independent reviewers using the National Heart, Lung, and Blood Institute tool for observational and cross-sectional studies [31]. This tool includes 14 items that are answered with “yes,” “no,” or “not applicable.” These will then be converted into a dichotomous rating (“yes” = 1, “no” and “not applicable” = 0) [17]. Every citation will receive a score by summing up all 14 items [17]. Poor, fair, and good study quality will correspond to a score of 0‑5, 6‑9, and 10‑14, respectively [17]. The scores will be added to the data collection form prior to the analyses. The results of the risk of bias assessment will be presented in a table. Occurring conflicts will be solved by consensus between the two reviewers (FS and EP). In the case of no consensus, a third reviewer (JG) will be consulted.

Following this process, the stringency index reported in the Oxford COVID-19 Government Response Tracker database at the respective periods the studies were conducted will be matched to the citations in the data collection form accordingly [32]. In case of missing data or differences in safety measures depending on the region of a country, we will gather further information by searching publicly available information, i.e., official governmental documents, newspaper articles, etc., and calculate the missing stringency index [32].

### Statistical analysis

The package “metafor” for R (R Foundation for Statistical Computing, Vienna, Austria) will be used to perform the meta-analysis and create meta-analytical forest plots [33]. Relevant information of the included studies (i.e., citation, subject characteristics, country, time of data collection, measurement tool, and study design) will be summarized and presented in a table. The standardized mean difference will be calculated and expressed as Hedges’s adjusted *g* with 95% confidence interval because PA will likely have been assessed using a variety of methods [34]. On condition of enough relevant studies being available, we will conduct random-effects meta-analyses to assess the overall effect of safety measures on (1) total PA and (2) sedentary time as well as LPA, MPA, and VPA or MVPA. In case there are not enough relevant studies available, a narrative, qualitative summary will be conducted.

The effect of heterogeneity (*I*^2^) will be calculated based on Cochrane’s *Q*, with a value of 0% indicating no heterogeneity and 100% substantial heterogeneity [35]. The degree of heterogeneity will be rated based on the proposed Cochrane categories [24]. Heterogeneity among the included studies will be investigated by a sensitivity analysis that takes the results of the risk of bias assessment into account.

A meta-regression will be performed to investigate the effect of the severity of the safety measures with the stringency index [32] as the independent variable and standardized mean difference in TPA as the dependent variable.

Furthermore, we will run subgroup analyses to examine the impact of self-reported (i.e., using questionnaires, interviews, diaries) vs. objectively assessed PA (i.e., through wearables, mobile applications, global positioning system), differences between age groups (i.e., using age tertiles), and the time passed after the declaration of the pandemic (i.e., with time passed transformed into categorical variables). However, for these analyses, at least three studies per group need to be available. Finally, the confidence in the cumulative body of evidence will be assessed using the GRADE guidelines [36].

To avoid the emerging of an ecological fallacy, no conclusions on individual-level effects will be drawn based on aggregated study-level results [24]. All results of the planned study will be interpreted with caution bearing in mind the issue of an ecological fallacy. Finally, the option to request individual-level data from the authors of relevant studies and subsequently perform an individual data meta-analysis will be reserved. This will depend on the number of studies after full-text screening and temporal resources.

## Discussion

This systematic review and meta-analysis will provide important knowledge about changes in PA patterns due to the enacted safety measures during the COVID-19 pandemic in the adult general population. The major novelty will be that it takes the severity of safety measures in the respective countries into account and includes relevant papers published within the last two years. This will create an important basis for public health policymaking as well as the estimation of potential health consequences for public health. Finally, we will identify gaps in the literature and highlight important areas of future research.

Concerning the strengths and weaknesses of this study, we believe that the inclusion of only studies that used quantitative measures of PA will improve the accuracy and applicability of the results even though this will result in the loss of some relevant studies. Secondarily, by additionally searching for gray literature, we aim to reduce the risk of publication bias. Searching the selected databases may lead to a potential exclusion of relevant articles that are not in these databases. Yet, we feel that the most relevant sources have been included in this protocol. Studies including only patients with acute SARS-nCOV-2 infection or that recovered from this infection will be excluded, to obtain a reliable picture of the effect of safety measures on PA patterns in the adult general population that was not directly affected by the virus. Since the literature search was already initiated using the previously presented search strategy, studies focusing on mask mandates will not explicitly be searched for. This poses a limitation. A further potential pitfall of the presented research questions may be large differences in effect sizes between countries and points in time. This will be addressed by meta-regression including a unit of safety measure severity in the model as previously described.

Any relevant study protocol amendments, as well as the study progress, will be documented on PROSPERO. The final manuscript will be submitted to a relevant peer-reviewed journal in this field for consideration for publication. Furthermore, results will be presented at international scientific conferences.

## Supplementary Information

**Additional file 1.** Completed PRISMA-P Checklist.

**Additional file 2.** Full search strategy for Medline (on Ovid), Web of Science (core collection), Scopus, L^.^OVE Coronavirus disease by Epistemonikos, and ProQuest Dissertations & Theses A&I (on proquest.com).

## Data Availability

Not applicable.

## Abbreviations

COVID-19: Coronavirus disease 2019
LPA: Light physical activity
MPA: Moderate physical activity
MVPA: Moderate-to-vigorous physical activity
PA: Physical activity
PROSPERO: International Prospective Register of Systematic Reviews database
SARS-nCOV-2: Severe acute respiratory syndrome coronavirus 2
TPA: Total physical activity
VPA: Vigorous physical activity
