# Supplementary material for: Protocol for a systematic review and meta-analysis of observational studies examining the impact of COVID-19 safety measures on physical activity patterns in adults
Source: Syst Rev. 2021 Oct 29;10:281. doi: 10.1186/s13643-021-01818-y (PMC8554735; doi:10.1186/s13643-021-01818-y)
Supplement: Supplementary file 2 — Additional file 2. Full search strategy for Medline (on Ovid), Web of Science (core collection), Scopus, L.OVE Coronavirus disease by Epistemonikos, and ProQuest Dissertations & Theses A&I (on proquest.com). [file 13643_2021_1818_MOESM2_ESM.pdf]

Additional file 2: Full search strategy

# Protocol for a Systematic Review and Meta-Analysis of Observational Studies Examining the Impact of COVID-19 Safety Measures on Physical Activity Patterns in Adults

Schwendinger, F.[1], Infanger, D.[1], Pococco, E.[2], Gander, J.[1], Hinrichs, T.[1] & Schmidt-Trucksäss, A.[1]

[1]Division of Sports and Exercise Medicine, Department of Sport, Exercise and Health, University of Basel, Birsstrasse 320 B, 4052 Basel, Switzerland

[2]Department of Sports Science, Medical Section, University of Innsbruck, Fürstenweg 185, 6020 Innsbruck, Austria

Corresponding author:

Arno Schmidt-Trucksäss (AST), arno.schmidt-trucksaess@unibas.ch; Tel: +41 (0) 61 2074741; Fax: +41 (0) 61 2074742; Department of Sport, Exercise and Health, University of Basel, Birsstrasse 320 B, 4052 Basel, Switzerland; ORCID: https://orcid.org/0000-0002-4662-3911

Fabian Schwendinger (FS), fabian.schwendinger@unibas.ch; ORCID: https://orcid.org/0000-0001-7795-1478

Denis Infanger (DI), denis.infanger@unibas.ch; ORCID: https://orcid.org/0000-0001-9028-7110

Elena Pococco (EP), elenapococco@yahoo.it; ORCID: https://orcid.org/0000-0002-3730-1961

Additional file 2: Full search strategy

Joséphine Gander (JG), josephine.gander@unibas.ch; ORCID: https://orcid.org/0000-0001-7774-7100

Timo Hinrichs (TH), timo.hinrichs@unibas.ch; ORCID: https://orcid.org/0000-0001-6200-307X

# Full Search Strategy

**Ovid Search String:**

**POPULATION 1**
Search strategy to exclude unwanted populations:

(Search strategy) NOT (exp animals/ NOT humans/)

**POPULATION 2**
Search strategy for COVID-19 (developed by Canadian Agency for Drugs and Technologies in Health (1)):

1. (covid-19 or SARS-CoV-2).sh
2. (coronavirus/ OR betacoronavirus/ OR coronavirus infections/) and (disease outbreaks/ OR epidemics/ OR pandemics/)
3. (nCoV* OR 2019nCoV OR 19nCoV OR COVID19* OR COVID OR SARS-COV-2 OR SARSCOV-2 OR SARSCOV2 OR Severe Acute Respiratory Syndrome Coronavirus 2 OR Severe Acute Respiratory Syndrome Corona Virus 2).ti,ab,kf,nm,ot,ox,rx,px.
4. ((new OR novel OR 19 OR 2019 OR Wuhan OR Hubei OR China OR Chinese) adj3 (coronavirus* OR corona virus* OR betacoronavirus* OR CoV OR HCoV)).ti,ab,kf,ot.
5. ((coronavirus* OR corona virus* OR betacoronavirus*) adj3 (pandemic* OR epidemic* OR outbreak* OR crisis)).ti,ab,kf,ot.
6. ((Wuhan OR Hubei) adj5 pneumonia).ti,ab,kf,ot.
7. OR/1-6

**INTERVENTION 1**
Search strategy for safety measures (developed by Canadian Agency for Drugs and Technologies in Health (1)):

1. Social Distance/ OR social isolation/
2. exp pandemics/pc or (pandemic* adj3 (prevention or control)).ti,ab.
3. ((social* OR physical* OR spatial* OR person* OR interperson*) adj1 distanc*).ti,ab,kf.
4. ((physical* OR social*) adj separat*).ti,ab,kf.
5. (lockdown* OR lock down* OR self quarantin* OR stay at home OR stay home OR remain* at home OR shelter* in place OR safety measure*).ti,ab,kf.
6. (home adj2 (isolate OR isolated OR isolati* OR quarantin* OR confin*)).ti,ab,kf.
7. (self isolat* OR household isolat* OR house hold isolat*).ti,ab,kf.
8. quarantine/ and (home OR self).ti,ab,kf.
9. ((gathering* OR public event* OR public activit* OR group event* OR group activit* OR mass event* OR large event* OR social event* OR social activit* OR nonessential activit* OR non essential activit* OR conference* OR sporting OR travel* OR public transit OR public transport* OR worship* OR (religio* adj2 (event* OR rite OR rites OR service* OR building* OR organization* OR organisation*)) OR church* OR synagogue* OR mosque* OR temple* OR night club

OR night clubs OR nightclub* OR concerts) adj3 (restrict* OR limit* OR cancel* OR reduc* OR postpon* OR post pon* OR ban OR bans OR banned OR banning OR suspend* OR suspension)).ti,ab,kf.

10. ((social OR contact OR contacts OR crowd*) adj2 (restrict* OR limit* OR reduc* OR mixing)).ti,ab,kf.
11. ((school* OR classroom* OR class room* OR educational OR universit* OR college* OR workplace* OR work place* OR worksite* OR work site* OR office OR offices OR business* OR restaurant* OR retail OR store OR stores OR public space* OR public area* OR communal space* OR communal area* OR venue* OR public transit OR public transport* OR worship* OR (religio* adj2 (event* OR rite OR rites OR service* OR building* OR organization* OR organisation*)) OR church* OR synagogue* OR mosque* OR temple* OR club OR clubs OR nightclub* OR sport OR sports OR park OR parks) adj3 (closure* OR close OR closed OR closing OR shutdown* OR shut down)).ti,ab,kf.
12. work closure*.ti,ab,kf.
13. (schools/ OR exp travel/) and (restrict* OR limit* OR cancel* OR reduc* OR postpon* OR post pon* OR ban OR bans OR banned OR banning OR suspend* OR suspension).ti,ab,kf.
14. ((school* OR class*) adj1 dismiss*).ti,ab,kf.
15. ((gathering* OR public event* OR public activit* OR group event* OR group activit* OR mass event* OR large event* OR social event* OR social activit* OR nonessential activit* OR non essential activit* OR conference* OR sporting OR public transit OR public transport* OR worship* OR (religio* adj2 (event* OR rite OR rites OR service* OR building* OR organization* OR organisation*)) OR church* OR synagogue* OR mosque* OR temple* OR nightclub* OR concerts OR school* OR classroom* OR class room* OR educational OR universit* OR college* OR workplace* OR work OR worksite* OR office OR offices OR business* OR restaurant* OR retail OR store OR stores OR public space* OR public area* OR communal space* OR communal area* OR venue* OR club OR clubs OR sport OR sports OR park OR parks) adj4 (reopen* OR re open* OR resum* OR restart* OR re start* OR return*)).ti,ab,kf.
16. (travel* adj3 (reopen* OR re open* OR resum* OR restart* OR re start*)).ti,ab,kf.
17. OR/1-16

**OUTCOME**

Search strategy for outcome:

1. exp exercise/ OR exp physical fitness/ OR exp sports/ OR exp running/ OR exp walking/ OR exp sedentary behavior/ OR exp leisure activities/
2. fitness trackers/ OR monitoring, ambulatory/
3. ((physical activity OR exercise OR physical fitness) adj4 (smartphone* OR mobile* OR cell phone* OR app OR application* OR measurement)).ti,ab,kf.
4. (sport* OR athletic* OR football OR gymnastic* OR soccer OR skating OR bicycling OR jogging OR running OR physical fitness OR motor activity OR physical inactiv* OR fitness tracker* OR activity tracker* OR ambulatory monitoring OR outpatient monitoring).ti,ab,kf.
5. (tv time OR television time OR computer use OR screen time OR screen viewing OR video gaming).ti,ab,kf.

6. (exercise* OR physical activit* OR active travel* OR walk* OR cycling OR bicycle OR sit OR sitting OR sedentary OR sedentari* OR acceleromet* OR step* count OR steps OR pedometer OR pedometry OR motion sensor* OR fitbit* OR activity monitor* OR wearable sensor* OR movement count OR activity bout* OR bouts of activity OR garmin* OR IPAQ OR international physical activity questionnaire OR GPAQ OR global physical activity questionnaire OR physical activity survey* OR physical activity questionnaire*).ti,ab,kf.
7. OR/1-6

Applied to full search: limit to yr=”2019-Current”

The Polyglot Search Translator was used to translate this search strategy for use in other relevant databases.(2) The translations were then evaluated manually by a medical librarian.

Additional file 2: Full search strategy

**Web of Science Core Collection Search String (on Web of Science):**

**POPULATION**

Search strategy for COVID-19 (modified from Canadian Agency for Drugs and Technologies in Health (1)):

1. TS=(nCoV* OR 2019nCoV OR 19nCoV OR COVID19* OR COVID OR SARS-COV-2 OR SARSCOV-2 OR SARSCOV2 OR “Severe Acute Respiratory Syndrome Coronavirus 2“ OR “Severe Acute Respiratory Syndrome Corona Virus 2”)
2. TS=((new OR novel OR “19” OR “2019” OR Wuhan OR Hubei OR China OR Chinese) near/3 (coronavirus* OR “corona virus*” OR betacoronavirus* OR CoV OR HCoV))
3. TS=((coronavirus* OR “corona virus*” OR betacoronavirus*) near/5 (pandemic* OR epidemic* OR outbreak* OR crisis))
4. TS=((Wuhan OR Hubei) near/5 pneumonia)
5. #1 OR #2 OR #3 OR #4

**INTERVENTION**

Search strategy for safety measures (modified from Canadian Agency for Drugs and Technologies in Health (1)):

1. TS=("Social Distance" OR "social isolation")
2. TS=(((physical* OR social*) NEAR/0 separat*))
3. TS=((lockdown* OR "lock down*" OR "self quarantin*" OR "stay at home" OR "stay home" OR "remain* at home" OR "shelter* in place" OR “safety measure*”))
4. TS=((home NEAR/2 (isolate OR isolated OR isolati* OR quarantin* OR confin*)))
5. TS=(("self isolat*" OR "household isolat*" OR "house hold isolat*"))
6. TS=(((gathering* OR "public event*" OR "public activit*" OR "group event*" OR "group activit*" OR "mass event*" OR "large event*" OR "social event*" OR "social activit*" OR "nonessential activit*" OR "non essential activit*" OR conference* OR sporting OR travel* OR "public transit" OR "public transport*" OR worship* OR (religio* NEAR/2 (event* OR rite OR rites OR service* OR building* OR organization* OR organisation*)) OR church* OR synagogue* OR mosque* OR temple* OR "night club" OR "night clubs" OR nightclub* OR concerts) NEAR/3 (restrict* OR limit* OR cancel* OR reduc* OR postpon* OR "post pon*" OR ban OR bans OR banned OR banning OR suspend* OR suspension)))
7. TS=(((social OR contact OR contacts OR crowd*) NEAR/2 (restrict* OR limit* OR reduc* OR mixing)))
8. TS=(((school* OR classroom* OR "class room*" OR educational OR universit* OR college* OR workplace* OR "work place*" OR worksite* OR "work site*" OR office OR offices OR business* OR restaurant* OR retail OR store OR stores OR "public space*" OR "public area*" OR "communal space*" OR "communal area*" OR venue* OR "public transit" OR "public transport*" OR worship* OR (religio* NEAR/2 (event* OR rite OR rites OR service* OR building* OR organization* OR organisation*)) OR church* OR synagogue* OR mosque* OR temple* OR club OR clubs OR nightclub* OR sport OR sports OR park OR parks) NEAR/3 (closure* OR close OR closed OR closing OR shutdown* OR "shut down")))
9. TS=("work closure*")

10. TS=((schools OR travel) AND (restrict* OR limit* OR cancel* OR reduc* OR postpon* OR "post pon*" OR ban OR bans OR banned OR banning OR suspend* OR suspension))
11. TS=(((gathering* OR "public event*" OR "public activit*" OR "group event*" OR "group activit*" OR "mass event*" OR "large event*" OR "social event*" OR "social activit*" OR "nonessential activit*" OR "non essential activit*" OR conference* OR sporting OR "public transit" OR "public transport*" OR worship* OR (religio* NEAR/2 (event* OR rite OR rites OR service* OR building* OR organization* OR organisation*)) OR church* OR synagogue* OR mosque* OR temple* OR nightclub* OR concerts OR school* OR classroom* OR "class room*" OR educational OR universit* OR college* OR workplace* OR work OR worksite* OR office OR offices OR business* OR restaurant* OR retail OR store OR stores OR "public space*" OR "public area*" OR "communal space*" OR "communal area*" OR venue* OR club OR clubs OR sport OR sports OR park OR parks) NEAR/4 (reopen* OR "re open*" OR resum* OR restart* OR "re start*" OR return*)))
12. TS=((travel* NEAR/3 (reopen* OR "re open*" OR resum* OR restart* OR "re start*")))
13. #1 OR #2 OR #3 OR #4 OR #5 OR #6 OR #7 OR #8 OR #9 OR #10 OR #11 OR #12 OR #13 OR #14 OR #15

**OUTCOME**

Search strategy for outcome:

1. TS=((("physical activity" OR exercise* OR sports OR "physical fitness") NEAR/4 (smartphone* OR mobile* OR "cell phone*" OR app OR application* OR measurement)))
2. TS=((exercise* OR "physical activit*" OR "active travel*" OR "leisure activit*" OR "walking activity" OR walk* OR walking OR run* OR cycling OR bicycle OR sit OR sitting OR sedentary OR accelerometer OR acceleromet* OR acceleration OR fitness tracker* OR activity tracker* OR ambulatory monitoring OR outpatient monitoring OR "step* count" OR steps OR pedometer OR pedometry OR "motion sensor*" OR fitbit* OR "activity monitor*" OR wearable* OR "wearable sensor*" OR "movement count" OR "activity bout*" OR "bouts of activity" OR garmin* OR IPAQ OR "international physical activity questionnaire" OR GPAQ OR "global physical activity questionnaire" OR "physical activity survey*" OR "physical activity questionnaire*" OR sport* OR athletic* OR football OR gymnastic* OR soccer OR skating OR bicycling OR jogging OR running OR motor activity OR physical inactiv*))
3. TS=("tv time" OR "television time" OR "computer use" OR "screen time" OR "screen viewing" OR "video gaming")
4. #1 OR #2 OR #3

Applied to full search: AND PY= 2019-3000

Additional file 2: Full search strategy

**L-OVE platform by Epistemonikos Search String (on L·OVE Coronavirus disease [COVID-19] "advanced search beta"):**

**POPULATION**
Search strategy to exclude unwanted populations:

1. NOT ((infant OR child OR adolescent) NOT (adult)) NOT (animals NOT humans)

**POPULATION 2**
This will not be searched for.

**INTERVENTION**
Search strategy for safety measures (modified from Canadian Agency for Drugs and Technologies in Health (1)):

1. ((social* OR physical* OR spatial* OR person* OR interperson*) AND distanc*)
2. ((physical* OR social*) AND separat*)
3. (lockdown* OR "lock down*" OR "self quarantin*" OR "stay at home" OR "stay home" OR "remain* at home" OR "shelter* in place" OR "safety measure*")
4. (home AND (isolate OR isolated OR isolati* OR quarantin* OR confin*))
5. ("self isolat*" OR "household isolat*" OR "house hold isolat*")
6. (home OR self) AND quarantine
7. ((gathering* OR "public event*" OR "public activit*" OR "group event*" OR "group activit*" OR "mass event*" OR "large event*" OR "social event*" OR "social activit*" OR "nonessential activit*" OR "non essential activit*" OR conference* OR sporting OR travel* OR "public transit" OR "public transport*" OR worship* OR (religio* AND (event* OR rite OR rites OR service* OR building* OR organization* OR organisation*)) OR church* OR synagogue* OR mosque* OR temple* OR "night club" OR "night clubs" OR nightclub* OR concerts) AND (restrict* OR limit* OR cancel* OR reduc* OR postpon* OR "post pon*" OR ban OR bans OR banned OR banning OR suspend* OR suspension))
8. ((social OR contact OR contacts OR crowd*) AND (restrict* OR limit* OR reduc* OR mixing))
9. ((school* OR classroom* OR "class room*" OR educational OR universit* OR college* OR workplace* OR "work place*" OR worksite* OR "work site*" OR office OR offices OR business* OR restaurant* OR retail OR store OR stores OR "public space*" OR "public area*" OR "communal space*" OR "communal area*" OR venue* OR "public transit" OR "public transport*" OR worship* OR (religio* AND (event* OR rite OR rites OR service* OR building* OR organization* OR organisation*)) OR church* OR synagogue* OR mosque* OR temple* OR club OR clubs OR nightclub* OR sport OR sports OR park OR parks) AND (closure* OR close OR closed OR closing OR shutdown* OR "shut down"))
10. "work closure*"
11. (schools OR travel) AND (restrict* OR limit* OR cancel* OR reduc* OR postpon* OR post pon* OR ban OR bans OR banned OR banning OR suspend* OR suspension)
12. ((school* OR class*) AND dismiss*)
13. ((gathering* OR "public event*" OR "public activit*" OR "group event*" OR "group activit*" OR "mass event*" OR "large event*" OR "social event*" OR "social activit*" OR "nonessential activit*" OR "non essential activit*" OR conference* OR

sporting OR “public transit” OR “public transport*” OR worship* OR (religio* AND (event* OR rite OR rites OR service* OR building* OR organization* OR organisation*)) OR church* OR synagogue* OR mosque* OR temple* OR nightclub* OR concerts OR school* OR classroom* OR “class room*” OR educational OR universit* OR college* OR workplace* OR work OR worksite* OR office OR offices OR business* OR restaurant* OR retail OR store OR stores OR “public space*” OR “public area*” OR “communal space*” OR “communal area*” OR venue* OR club OR clubs OR sport OR sports OR park OR parks) AND (reopen* OR “re open*” OR resum* OR restart* OR “re start*” OR return*))

14. (travel* AND (reopen* OR “re open*” OR resum* OR restart* OR “re start*”))
15. OR/1-14

**OUTCOME**
Search strategy for outcome:

exercise OR “physical activit*” OR “leisure activit*” OR “physical fitness” OR sports OR locomotion OR “motor activity” OR accelerometer OR acceleromet* OR acceleration OR “step* count” OR steps OR pedometer OR pedometry OR “motion sensor*” OR fitbit* OR “fitness trackers” OR “activity monitor*” OR wearable* OR “wearable sensor*” OR “movement count” OR “activity bout*” OR “bouts of activity” OR “active travel*” OR garmin* OR “walking activity” OR walk OR walking OR cycling OR bicycle OR sit* OR sitting OR “sedentary behavior” OR sedentary OR sedentari* OR ((“physical activity” OR exercise OR “physical fitness”) AND (smartphone* OR mobile* OR “cell phone*” OR app OR application* OR measurement)) OR IPAQ OR “international physical activity questionnaire” OR GPAQ OR “global physical activity questionnaire” OR “physical activity survey*” OR “physical activity questionnaire*” OR sport* OR athletic* OR football OR gymnastic* OR soccer OR skating OR bicycling OR jogging OR running OR “physical fitness” OR “motor activity” OR “physical inactiv*” OR “fitness tracker*” OR “activity tracker*” OR “ambulatory monitoring” OR “outpatient monitoring” OR “tv time” OR “television time” OR “computer use” OR “screen time” OR “screen viewing” OR “video gaming”

Applied to full search: limit publication year to ”2019 -Current”

Additional file 2: Full search strategy

**Scopus Search String (on scopus.com):**

**POPULATION**
Search strategy for COVID-19 (developed by Canadian Agency for Drugs and Technologies in Health (1)):

((KEY(coronavirus OR betacoronavirus OR “coronavirus infections”) AND KEY (“disease outbreaks” OR epidemics OR pandemics)) OR (TITLE-ABS-KEY (ncov* OR 2019ncov OR 19ncov OR covid19* OR covid OR sars-cov-2 OR sars-cov2 OR sarscov-2 OR sarscov2 OR “Severe Acute Respiratory Syndrome Coronavirus 2” OR “Severe Acute Respiratory Syndrome Corona Virus 2”)) OR (TITLE-ABS-KEY ((new W/3 coronavirus*) OR (new W/3 “corona virus*”) OR (new W/3 betacoronavirus*) OR (new W/3 cov ) OR (new W/3 hcov) OR (novel W/3 coronavirus*) OR (novel W/3 “corona virus*”) OR (novel W/3 betacoronavirus*) OR (novel W/3 cov) OR (novel W/3 hcov) OR (19 W/3 coronavirus*) OR (19 W/3 “corona virus*”) OR (19 W/3 betacoronavirus*) OR (19 W/3 cov) OR (19 W/3 hcov) OR (2019 W/3 coronavirus*) OR (2019 W/3 “corona virus*”) OR (2019 W/3 betacoronavirus*) OR (2019 W/3 cov) OR (2019 W/3 hcov) OR ( wuhan W/3 coronavirus*) OR (wuhan W/3 “corona virus*”) OR (wuhan W/3 betacoronavirus*) OR (wuhan W/3 cov) OR (wuhan W/3 hcov) OR (hubei W/3 coronavirus*) OR (hubei W/3 “corona virus*”) OR (hubei W/3 betacoronavirus*) OR (hubei W/3 cov) OR (hubei W/3 hcov) OR (china W/3 coronavirus*) OR (china W/3 “corona virus*”) OR (china W/3 betacoronavirus*) OR (china W/3 cov) OR (china W/3 hcov) OR (chinese W/3 coronavirus*) OR (chinese W/3 “corona virus*”) OR (chinese W/3 betacoronavirus*) OR (chinese W/3 cov) OR (chinese W/3 hcov))) OR (TITLE-ABS-KEY((coronavirus* W/3 pandemic*) OR (coronavirus* W/3 epidemic*) OR (coronavirus* W/3 outbreak*) OR (coronavirus* W/3 crisis) OR (“corona virus*” W/3 pandemic*) OR (“corona virus*” W/3 epidemic*) OR (“corona virus*” W/3 outbreak*) OR (“corona virus*” W/3 crisis) OR (betacoronavirus* W/3 pandemic*) OR (betacoronavirus* W/3 epidemic*) OR (betacoronavirus* W/3 outbreak*) OR (betacoronavirus* W/3 crisis))) OR (TITLE-ABS-KEY((wuhan W/5 pneumonia) OR (hubei W/5 pneumonia))))

**INTERVENTION**
Search strategy for safety measures (modified from Canadian Agency for Drugs and Technologies in Health (1)):

1. INDEXTERMS("Social Distance") OR INDEXTERMS("social isolation")
2. ((TITLE-ABS-KEY("social*") OR TITLE-ABS-KEY("physical*") OR TITLE-ABS-KEY("spatial*") OR TITLE-ABS-KEY("person*") OR TITLE-ABS-KEY("interperson*")) W/1 TITLE-ABS-KEY("distanc*"))
3. ((TITLE-ABS-KEY("physical*") OR TITLE-ABS-KEY("social*")) W/1 TITLE-ABS-KEY("separat*"))
4. (TITLE-ABS-KEY("lockdown*") OR TITLE-ABS-KEY("lock down*") OR TITLE-ABS-KEY("self quarantin*") OR TITLE-ABS-KEY("stay at home") OR TITLE-ABS-KEY("stay home") OR TITLE-ABS-KEY("remain* at home") OR TITLE-ABS-KEY("shelter* in place") OR TITLE-ABS-KEY("safety measure*"))
5. (TITLE-ABS-KEY("home") W/2 (TITLE-ABS-KEY("isolate") OR TITLE-ABS-KEY("isolated") OR TITLE-ABS-KEY("isolati*") OR TITLE-ABS-KEY("quarantin*") OR TITLE-ABS-KEY("confin*")))
6. (TITLE-ABS-KEY("self isolat*") OR TITLE-ABS-KEY("household isolat*") OR TITLE-ABS-KEY("house hold isolat*"))

7. INDEXTERMS("quarantine") AND (TITLE-ABS-KEY("home") OR TITLE-ABS-KEY("self"))
8. ((TITLE-ABS-KEY("gathering*") OR TITLE-ABS-KEY("public event*") OR TITLE-ABS-KEY("public activit*") OR TITLE-ABS-KEY("group event*") OR TITLE-ABS-KEY("group activit*") OR TITLE-ABS-KEY("mass event*") OR TITLE-ABS-KEY("large event*") OR TITLE-ABS-KEY("social event*") OR TITLE-ABS-KEY("social activit*") OR TITLE-ABS-KEY("nonessential activit*") OR TITLE-ABS-KEY("non essential activit*") OR TITLE-ABS-KEY("conference*") OR TITLE-ABS-KEY("sporting") OR TITLE-ABS-KEY("travel*") OR TITLE-ABS-KEY("public transit") OR TITLE-ABS-KEY("public transport*") OR TITLE-ABS-KEY("worship*") OR (TITLE-ABS-KEY("religio*") W/2 (TITLE-ABS-KEY("event*") OR TITLE-ABS-KEY("rite") OR TITLE-ABS-KEY("rites") OR TITLE-ABS-KEY("service*") OR TITLE-ABS-KEY("building*") OR TITLE-ABS-KEY("organization*") OR TITLE-ABS-KEY("organisation*"))) OR TITLE-ABS-KEY("church*") OR TITLE-ABS-KEY("synagogue*") OR TITLE-ABS-KEY("mosque*") OR TITLE-ABS-KEY("temple*") OR TITLE-ABS-KEY("night club") OR TITLE-ABS-KEY("night clubs") OR TITLE-ABS-KEY("nightclub*") OR TITLE-ABS-KEY("concerts")) W/3 (TITLE-ABS-KEY("restrict*") OR TITLE-ABS-KEY("limit*") OR TITLE-ABS-KEY("cancel*") OR TITLE-ABS-KEY("reduc*") OR TITLE-ABS-KEY("postpon*") OR TITLE-ABS-KEY("post pon*") OR TITLE-ABS-KEY("ban") OR TITLE-ABS-KEY("bans") OR TITLE-ABS-KEY("banned") OR TITLE-ABS-KEY("banning") OR TITLE-ABS-KEY("suspend*") OR TITLE-ABS-KEY("suspension")))
9. ((TITLE-ABS-KEY("social") OR TITLE-ABS-KEY("contact") OR TITLE-ABS-KEY("contacts") OR TITLE-ABS-KEY("crowd*")) W/2 (TITLE-ABS-KEY("restrict*") OR TITLE-ABS-KEY("limit*") OR TITLE-ABS-KEY("reduc*") OR TITLE-ABS-KEY("mixing")))
10. ((TITLE-ABS-KEY("school*") OR TITLE-ABS-KEY("classroom*") OR TITLE-ABS-KEY("class room*") OR TITLE-ABS-KEY("educational") OR TITLE-ABS-KEY("universit*") OR TITLE-ABS-KEY("college*") OR TITLE-ABS-KEY("workplace*") OR TITLE-ABS-KEY("work place*") OR TITLE-ABS-KEY("worksite*") OR TITLE-ABS-KEY("work site*") OR TITLE-ABS-KEY("office") OR TITLE-ABS-KEY("offices") OR TITLE-ABS-KEY("business*") OR TITLE-ABS-KEY("restaurant*") OR TITLE-ABS-KEY("retail") OR TITLE-ABS-KEY("store") OR TITLE-ABS-KEY("stores") OR TITLE-ABS-KEY("public space*") OR TITLE-ABS-KEY("public area*") OR TITLE-ABS-KEY("communal space*") OR TITLE-ABS-KEY("communal area*") OR TITLE-ABS-KEY("venue*") OR TITLE-ABS-KEY("public transit") OR TITLE-ABS-KEY("public transport*") OR TITLE-ABS-KEY("worship*") OR (TITLE-ABS-KEY("religio*") W/2 (TITLE-ABS-KEY("event*") OR TITLE-ABS-KEY("rite") OR TITLE-ABS-KEY("rites") OR TITLE-ABS-KEY("service*") OR TITLE-ABS-KEY("building*") OR TITLE-ABS-KEY("organization*") OR TITLE-ABS-KEY("organisation*"))) OR TITLE-ABS-KEY("church*") OR TITLE-ABS-KEY("synagogue*") OR TITLE-ABS-KEY("mosque*") OR TITLE-ABS-KEY("temple*") OR TITLE-ABS-KEY("club") OR TITLE-ABS-KEY("clubs") OR TITLE-ABS-KEY("nightclub*") OR TITLE-ABS-KEY("sport") OR TITLE-ABS-KEY("sports") OR TITLE-ABS-KEY("park") OR TITLE-ABS-KEY("parks")) W/3 (TITLE-ABS-KEY("closure*") OR TITLE-ABS-KEY("close") OR TITLE-ABS-

KEY("closed") OR TITLE-ABS-KEY("closing") OR TITLE-ABS-KEY("shutdown*") OR TITLE-ABS-KEY("shut down")))

11. TITLE-ABS-KEY("work closure*")
12. (INDEXTERMS("schools") OR INDEXTERMS("travel")) AND (TITLE-ABS-KEY("restrict*") OR TITLE-ABS-KEY("limit*") OR TITLE-ABS-KEY("cancel*") OR TITLE-ABS-KEY("reduc*") OR TITLE-ABS-KEY("postpon*") OR TITLE-ABS-KEY("post pon*") OR TITLE-ABS-KEY("ban") OR TITLE-ABS-KEY("bans") OR TITLE-ABS-KEY("banned") OR TITLE-ABS-KEY("banning") OR TITLE-ABS-KEY("suspend*") OR TITLE-ABS-KEY("suspension"))
13. ((TITLE-ABS-KEY("school*") OR TITLE-ABS-KEY("class*")) W/1 TITLE-ABS-KEY("dismiss*"))
14. ((TITLE-ABS-KEY("gathering*") OR TITLE-ABS-KEY("public event*") OR TITLE-ABS-KEY("public activit*") OR TITLE-ABS-KEY("group event*") OR TITLE-ABS-KEY("group activit*") OR TITLE-ABS-KEY("mass event*") OR TITLE-ABS-KEY("large event*") OR TITLE-ABS-KEY("social event*") OR TITLE-ABS-KEY("social activit*") OR TITLE-ABS-KEY("nonessential activit*") OR TITLE-ABS-KEY("non essential activit*") OR TITLE-ABS-KEY("conference*") OR TITLE-ABS-KEY("sporting") OR TITLE-ABS-KEY("public transit") OR TITLE-ABS-KEY("public transport*") OR TITLE-ABS-KEY("worship*") OR (TITLE-ABS-KEY("religio*") W/2 (TITLE-ABS-KEY("event*") OR TITLE-ABS-KEY("rite") OR TITLE-ABS-KEY("rites") OR TITLE-ABS-KEY("service*") OR TITLE-ABS-KEY("building*") OR TITLE-ABS-KEY("organization*") OR TITLE-ABS-KEY("organisation*"))) OR TITLE-ABS-KEY("church*") OR TITLE-ABS-KEY("synagogue*") OR TITLE-ABS-KEY("mosque*") OR TITLE-ABS-KEY("temple*") OR TITLE-ABS-KEY("nightclub*") OR TITLE-ABS-KEY("concerts") OR TITLE-ABS-KEY("school*") OR TITLE-ABS-KEY("classroom*") OR TITLE-ABS-KEY("class room*") OR TITLE-ABS-KEY("educational") OR TITLE-ABS-KEY("universit*") OR TITLE-ABS-KEY("college*") OR TITLE-ABS-KEY("workplace*") OR TITLE-ABS-KEY("work") OR TITLE-ABS-KEY("worksite*") OR TITLE-ABS-KEY("office") OR TITLE-ABS-KEY("offices") OR TITLE-ABS-KEY("business*") OR TITLE-ABS-KEY("restaurant*") OR TITLE-ABS-KEY("retail") OR TITLE-ABS-KEY("store") OR TITLE-ABS-KEY("stores") OR TITLE-ABS-KEY("public space*") OR TITLE-ABS-KEY("public area*") OR TITLE-ABS-KEY("communal space*") OR TITLE-ABS-KEY("communal area*") OR TITLE-ABS-KEY("venue*") OR TITLE-ABS-KEY("club") OR TITLE-ABS-KEY("clubs") OR TITLE-ABS-KEY("sport") OR TITLE-ABS-KEY("sports") OR TITLE-ABS-KEY("park") OR TITLE-ABS-KEY("parks")) W/4 (TITLE-ABS-KEY("reopen*") OR TITLE-ABS-KEY("re open*") OR TITLE-ABS-KEY("resum*") OR TITLE-ABS-KEY("restart*") OR TITLE-ABS-KEY("re start*") OR TITLE-ABS-KEY("return*")))
15. (TITLE-ABS-KEY("travel*") W/3 (TITLE-ABS-KEY("reopen*") OR TITLE-ABS-KEY("re open*") OR TITLE-ABS-KEY("resum*") OR TITLE-ABS-KEY("restart*") OR TITLE-ABS-KEY("re start*")))
16. #1 OR #2 OR #3 OR #4 OR #5 OR #6 OR #7 OR #8 OR #9 OR #10 OR #11 OR #12 OR #13 OR #14 OR #15

**OUTCOME**

Search strategy for outcome:

Additional file 2: Full search strategy

1. INDEXTERMS("exercise") OR INDEXTERMS("physical fitness") OR INDEXTERMS("sports") OR INDEXTERMS("bicycling") OR INDEXTERMS("walking") OR INDEXTERMS("leisure activities") OR INDEXTERMS("motor activity") OR INDEXTERMS("sedentary behavior")
2. INDEXTERMS("fitness trackers") OR INDEXTERMS("monitoring, ambulatory")
3. ((TITLE-ABS-KEY("physical activity") OR TITLE-ABS-KEY("exercise") OR TITLE-ABS-KEY("physical fitness")) W/4 (TITLE-ABS-KEY("smartphone*") OR TITLE-ABS-KEY("mobile*") OR TITLE-ABS-KEY("cell phone*") OR TITLE-ABS-KEY("app") OR TITLE-ABS-KEY("application*") OR TITLE-ABS-KEY("measurement")))
4. (TITLE-ABS-KEY("exercise") OR TITLE-ABS-KEY("physical activit*") OR TITLE-ABS-KEY("active travel*") OR TITLE-ABS-KEY("walking activity") OR TITLE-ABS-KEY("walk*") OR TITLE-ABS-KEY("walking") OR TITLE-ABS-KEY("cycling") OR TITLE-ABS-KEY("bicycle") OR TITLE-ABS-KEY("sit") OR TITLE-ABS-KEY("sitting") OR TITLE-ABS-KEY("sedentary") OR TITLE-ABS-Key("sedentari*")OR TITLE-ABS-KEY("accelerometer") OR TITLE-ABS-KEY("acceleromet*") OR TITLE-ABS-KEY("acceleration") OR TITLE-ABS-KEY("step* count") OR TITLE-ABS-KEY("steps") OR TITLE-ABS-KEY("pedometer") OR TITLE-ABS-KEY("pedometry") OR ((TITLE-ABS-KEY("physical activity") OR TITLE-ABS-KEY("exercise") OR TITLE-ABS-KEY("physical fitness")) W/7 (TITLE-ABS-KEY("armband*") OR TITLE-ABS-KEY("arm band*") OR TITLE-ABS-KEY("wristband") OR TITLE-ABS-KEY("wrist band"))) OR TITLE-ABS-KEY("motion sensor*") OR TITLE-ABS-KEY("fitbit*") OR TITLE-ABS-KEY("activity monitor*") OR TITLE-ABS-KEY("wearable*") OR TITLE-ABS-KEY("wearable sensor*") OR TITLE-ABS-KEY("movement count") OR TITLE-ABS-KEY("activity bout*") OR TITLE-ABS-KEY("bouts of activity") OR TITLE-ABS-KEY("garmin*") OR TITLE-ABS-KEY("IPAQ") OR TITLE-ABS-KEY("international physical activity questionnaire") OR TITLE-ABS-KEY("GPAQ") OR TITLE-ABS-KEY("global physical activity questionnaire") OR TITLE-ABS-KEY("physical activity survey*") OR TITLE-ABS-KEY("physical activity questionnaire*")) OR TITLE-ABS-KEY TITLE-ABS-KEY("tv time") OR TITLE-ABS-KEY("television time") OR TITLE-ABS-KEY("computer use") OR TITLE-ABS-KEY("screen time") OR TITLE-ABS-KEY("screen viewing") OR TITLE-ABS-KEY("video gaming")
5. #1 OR #2 OR #3 OR #4

Applied to full search: AND PUBYEAR AFT 2019

Additional file 2: Full search strategy

**Grey Literature Search String (on proquest.com Dissertations & Theses A&I):**

**POPULATION**
Search strategy for COVID-19:

Coronavirus; COVID-19; SARSnCOV-2; Severe Acute Respiratory Syndrome 2

**INTERVENTION**
Search strategy for COVID-19:

Safety measures; quarantine; lockdown; confinement; shutdown

**OUTCOME**
Search strategy for outcome:

Physical activity; physical inactivity; step; sports; exercise; mobility

Applied to full search: AND Publication date 2019-present

Additional file 2: Full search strategy

## References

1. Canadian Agency for Drugs and Technologies in Health. CADTH COVID-19 Search Strings: Canadian Agency for Drugs and Technologies in Health. https://covid.cadth.ca/literature-searching-tools/cadth-covid-19-search-strings/#covid-19-medline. Accessed 25 Feb 2021.
2. Clark JM, Sanders S, Carter M, Honeyman D, Cleo G, Auld Y, et al. Improving the translation of search strategies using the Polyglot Search Translator: a randomized controlled trial. J Med Libr Assoc. 2020;108(2):195-207.
